# Supplementary material for: The assassin bug Pristhesancus plagipennis produces two distinct venoms in separate gland lumens
Source: Nat Commun. 2018 Feb 22;9:755. doi: 10.1038/s41467-018-03091-5 (PMC5823883; doi:10.1038/s41467-018-03091-5)
Supplement: Supplementary file 1 — Supplementary Information [file 41467_2018_3091_MOESM1_ESM.pdf]

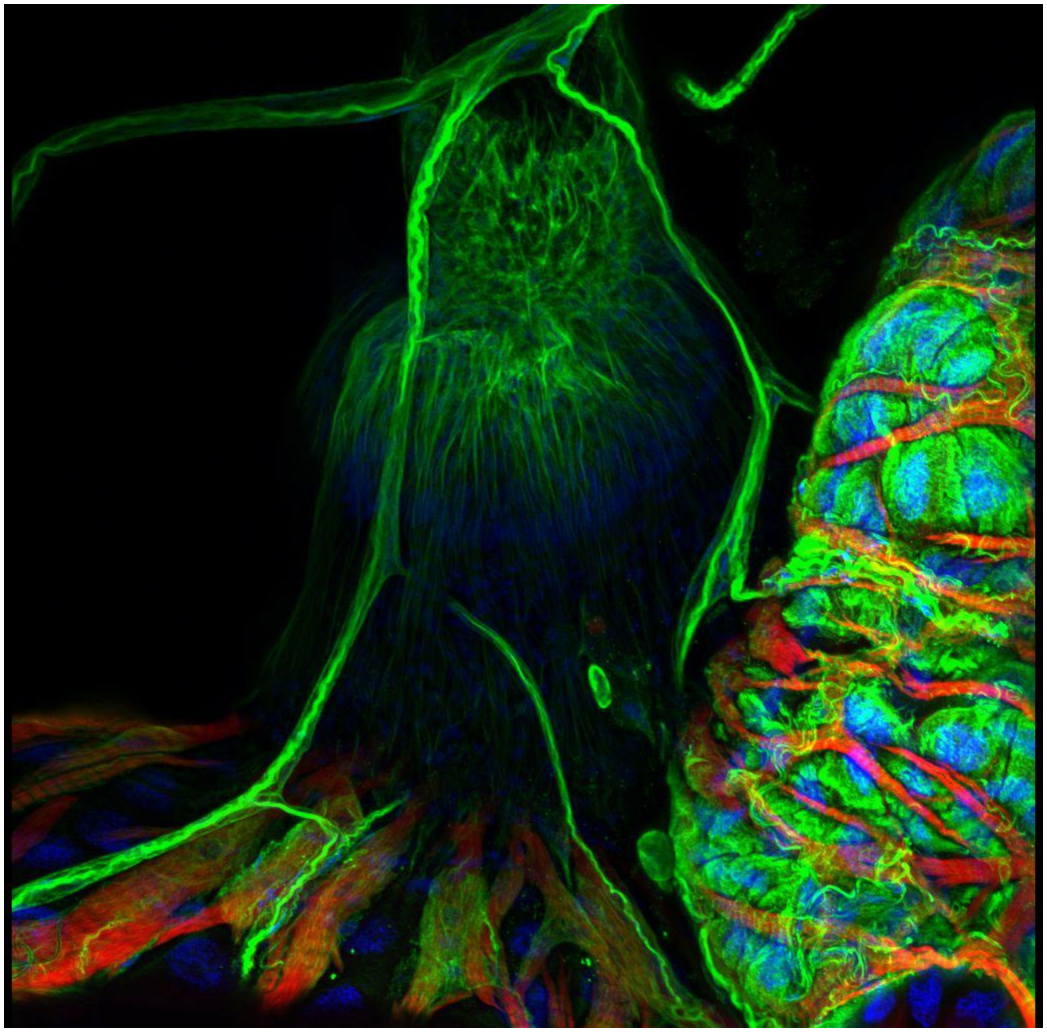

Supplementary Figure 1. Enlarged, unannotated version of Fig. 1j, right panel.

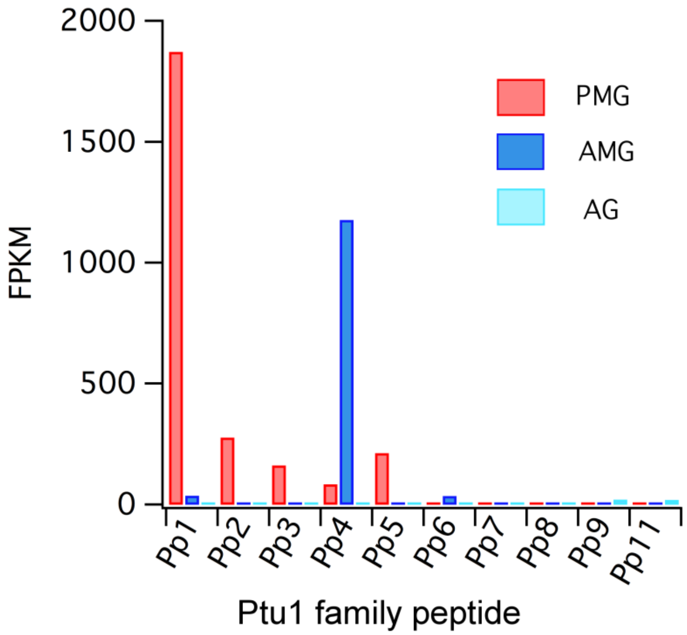

Supplementary Figure 2. Comparison of Ptu1 family peptide expression levels in each compartment of *P. plagipennis* venom glands.

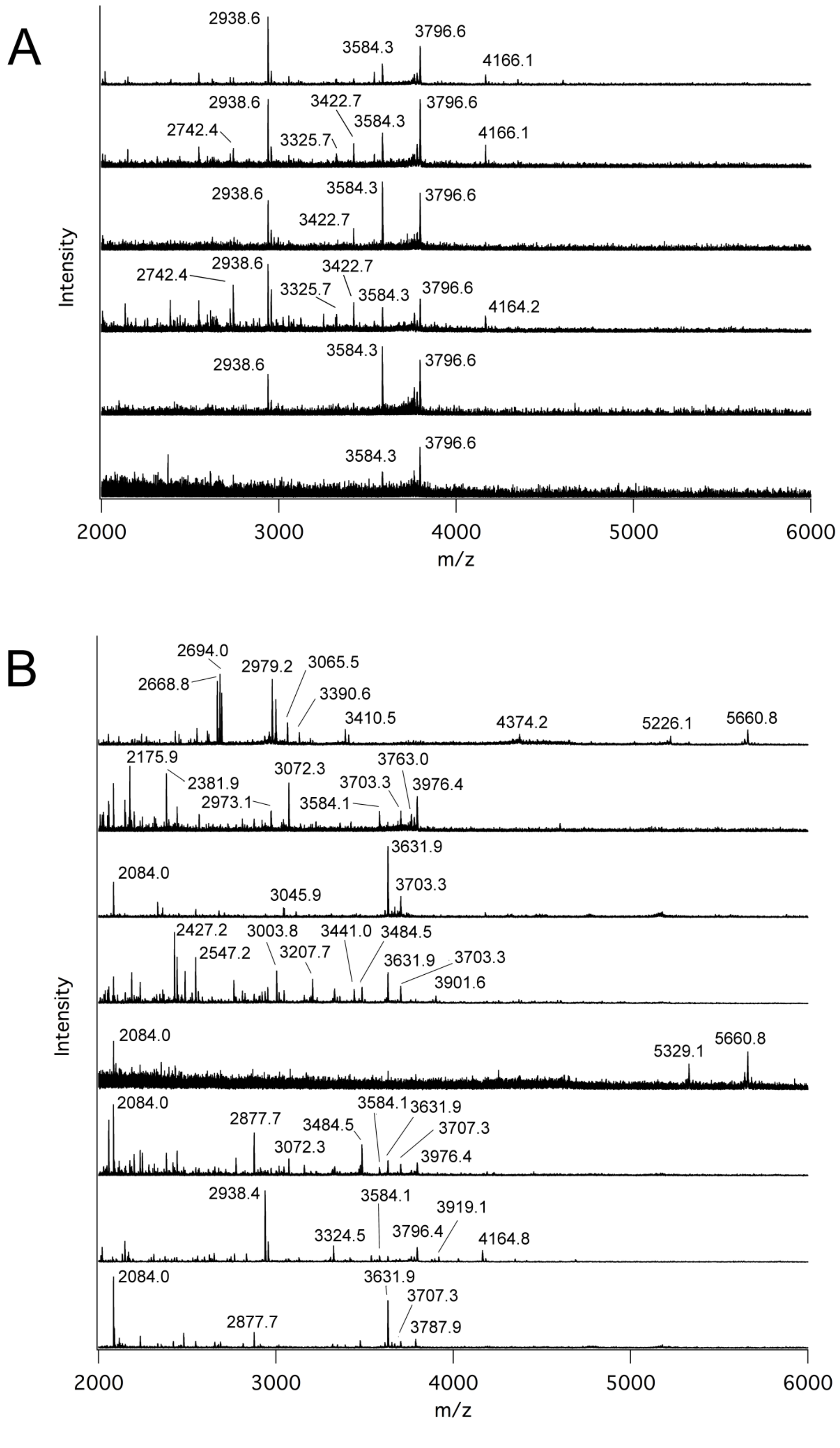

Supplementary Figure 3. High variability of mass spectra of venom samples obtained by harassment. Each sample was obtained on the same day from a different adult sibling housed in the same conditions.

Supplementary Table 1: Criteria for classifying proteins into groups based on primary structure characteristics.

Class/family	Criteria for inclusion
Protease	Protease domain (Pfam Trypsin, PF00089; Trypsin_2, PF13365; Astacin, PF01400; Peptidase_C1, PF0112) determined by HMMER ($E < 0.05$)
Hemolysin-like	BLAST homology ($E < 0.001$) to GenBank Accessions ABR27902, ABR27906, ABR27952 or another *P. plagipennis* protein in this group
Redulysin	BLAST homology ($E < 0.001$) to GenBank Accessions AAL82381, ADN29793, AQM58378, or another *P. plagipennis* protein in this group
Venom family 1	BLAST homology ($E < 0.001$) to GenBank Accessions AQM58355, AQM58356 or AQM58357, or another *P. plagipennis* protein in this group
Venom family 2	BLAST homology ($E < 0.001$) to GenBank Accessions AQM58358 through AQM58363 or another *P. plagipennis* protein in this group
CUB	CUB domain (Pfam 00431) determined by HMMER ($E < 0.05$) and/or BLAST homology ($E < 0.001$) to another *P. plagipennis* protein in this group; terminating in stop codon and lacking S1 protease domain; < 200 amino acids
Cystatin	Cystatin domain (Pfam 00031) determined by HMMER (E < 0.05) and/or BLAST homology ($E < 0.001$) to another *P. plagipennis* protein in this group
Kazal	Kazal domain (Kazal_1, PF00050 or Kazal_2, PF07648) determined by HMMER ($E < 0.05$) and/or BLAST homology ($E < 0.001$) to another *P. plagipennis* protein in this group
Pheremone/Odorant	Pheremone/odorant binding domain (Pfam 01395) detected by HMMER ($E < 0.05$) or BLAST homology ($E < 0.001$) to another *P. plagipennis* protein in this group
Ptu1 family peptide	Mature length < 60 amino acids with six cysteine residues in the arrangement C-C-CC-C-C, where dashes indicate additional loops of up to 20 non-cysteine residues
Nutrient	BLAST homology ($E < 0.001$) to a protein with a known function of nutrient binding or transport, e.g. transferrin
Other enzyme	BLAST homology ($E < 0.001$) to a protein with a known enzymatic but not proteolytic activity, e.g. kinase
Other	BLAST homology ($E < 0.001$) to a protein with a known non-enzymatic function, e.g. gelsolin
Uncharacterised	BLAST homology ($E < 0.001$) to other *P. plagipennis* proteins previously reported, but without putative function
Unknown	No detectable homology at BLAST homology ($E < 0.001$) or HMMER hits against Pfam ($E < 0.05$)
