# Supplementary material for: The assassin bug Pristhesancus plagipennis produces two distinct venoms in separate gland lumens
Source: Nat Commun. 2018 Feb 22;9:755. doi: 10.1038/s41467-018-03091-5 (PMC5823883; doi:10.1038/s41467-018-03091-5)
Supplement: Supplementary file 3 — Description of Additional Supplementary Files [file 41467_2018_3091_MOESM3_ESM.pdf]

Description of Additional Supplementary Files

File Name: Supplementary Data 1
Description: RNA-Seq analysis of venom gland transcription. Sheet A, Predicted secreted proteins in each compartment of *Pristhesancus plagipennis* venom glands, with sequence annotation and expression levels. Sheet B, Kruskal-Wallis tests of significance in transcript abundances between compartments.

File Name: Supplementary Data 2
Description: Proteomic analysis of accumulated protein content of venom glands. Sheets A–D, Identification and quantification of proteins in the venom glands of one adult. Protein identification has been performed against pooled open reading frames encoded by all gland compartments. Sheets E–M, Investigation of variability in proteins accumulated in each gland lumen. Protein identification has been performed by Paragon search of LC-MS/MS data against all venom proteins identified in this study.

File Name: Supplementary Data 3
Description: Venom protein content depends on harvesting method. Sheets A–B, All proteins detected in venom harvested by harassment (Sheet A) or electrostimulation (Sheet B) according to pooled Paragon search. Sheets C–R, results of Paragon searches for individual venom samples obtained from different individuals. Sheets C–J, eight venom samples from different individuals harvested by harassment; Sheets K–P, six venom samples from different individuals harvested by electrostimulation.

File Name: Supplementary Data 4
Description: Data used for correlations between venom composition and transcript abundance in each gland. Sheets A–B, Kruskal-Wallis test of expression levels of proteins detected only in venom harvested by electrostimulation (Sheet A) or harassment (Sheet B). Sheets C–D, Correlation of transcript expression levels in each compartment with protein abundance in venom obtained by electrostimulation (Sheet C; quantitation data obtained from electrostimulation venom sample 1 is shown in Supplementary Data S3, Sheet K) and harassment (Sheet D; quantitation data obtained from harassment venom sample 1 shown in Supplementary Data S3, Sheet C).

File Name: Supplementary Movie 1
Description: Animation showing 3D structure of the hilus and surrounding structures. This video was generated from a z-stack of DAPI staining images.

File Name: Supplementary Movie 2
Description: Venom expulsion with and without electrostimulation. Note the small quantity of venom is elicited by placing the proboscis into the collecting tip, though this is later reabsorbed (presumably ingested). Electrostimulation elicits a greater quantity of venom.

File Name: Supplementary Movie 3
Description: Venom harvesting by gentle harassment of a restrained bug without electrostimulation.

File Name: Supplementary Movie 4
Description: Harassment in the absence of a physical restraint shows characteristic defensive posturing.

File Name: Supplementary Movie 5
Description: Venom obtained by electrostimulation potently inhibits escape behaviour of crickets.
